# Impaired Contracture of 3D Collagen Constructs by Fibronectin-Deficient Murine Fibroblasts

**DOI:** 10.3389/fphys.2019.00166

**Published:** 2019-03-05

**Authors:** Joël Beyeler, Christos Katsaros, Matthias Chiquet

**Affiliations:** Laboratory for Oral Molecular Biology, Department of Orthodontics and Dentofacial Orthopedics, School of Dental Medicine, University of Bern, Bern, Switzerland

**Keywords:** fibronectin, alpha5-integrin, collagen, collagen contraction, collagen contracture, fibroblasts

## Abstract

Fibronectin (FN) is an extracellular matrix glycoprotein that is abundantly expressed by fibroblasts in contracting wounds, where it mediates cell adhesion, migration and proliferation. FN also efficiently binds to collagen. Therefore, we and others hypothesized that FN and its cellular receptor integrin α_5_β_1_ might be involved in collagen matrix contracture by acting as linkers. However, there are conflicting reports on this issue. Moreover, several publications suggest an important role of collagen-binding integrin receptors α_2_β_1_ and α_11_β_1_ in collagen matrix contracture. Therefore, the aim of the present study was to determine the contributions of FN–integrin α_5_β_1_ interactions relative to those of collagen receptors α_2_β_1_ and α_11_β_1_ in this process. To assess the role of cellular FN directly, we employed FN-deficient mouse fibroblasts, subjected them to a collagen gel contracture assay *in vitro*, and compared them to their wildtype counterparts. Exogenous FN was removed from serum-containing medium. For dissecting the role of major collagen receptors, we used two FN-deficient mouse fibroblast lines that both possess integrin α_5_β_1_ but differ in their collagen-binding integrins. Embryo-derived FN-null fibroblasts, which express α_11_- but no α_2_-integrin, barely spread and tended to cluster on collagen gels. Moreover, FN-null fibroblasts required exogenously added FN to assemble α_5_β_1_-integrin in fibrillar adhesion contacts, and to contract collagen matrices. In contrast, postnatal kidney fibroblasts were found to express α_2_- but barely α_11_-integrin. When FN expression was suppressed in these cells by shRNA transfection, they were able to spread on and partially contract collagen gels in the absence of exogenous FN. Also in this case, however, collagen contracture was stimulated by adding FN to the medium. Antibody to integrin α_5_β_1_ or RGD peptide completely abolished collagen contracture by FN-deficient fibroblasts stimulated by FN addition. We conclude that although collagen-binding integrins (especially α_2_β_1_) can mediate contracture of fibrillar collagen gels by murine fibroblasts to some extent, full activity is causally linked to the presence of pericellular FN and its receptor integrin α_5_β_1_.

## Introduction

Fibronectin (FN) is the prototype of an extracellular matrix (ECM) glycoprotein mediating cell adhesion. It is usually found as dimer of two nearly identical subunits (230–270 kDa) that are disulfide-linked at their C-terminus. Cells secreting FN assemble it into an irregular fibrillar network; a process that depends on integrins (primarily α_5_β_1_) and RhoA/ROCK-mediated actin contractility in epithelial and mesenchymal cells (Zhong et al., 1998; Fernandez-Sauze et al., 2009). During matrix maturation, the assembled FN fibrils can cluster into thicker bundles, forming a major constituent of the pericellular matrix (Schwarzbauer, 1991; Singh et al., 2010). Besides mediating cell adhesion, migration and RhoA-dependent responses to mechanical strain (Pankov and Yamada, 2002; Lutz et al., 2010), this meshwork appears to act as a scaffold for other ECM components: For example, fibrillin microfibril assembly (Kinsey et al., 2008), collagen fiber deposition (Sottile et al., 2007) and incorporation of fibrin into the ECM during tissue repair (Pereira and Simpson-Haidaris, 2001) depend on pericellular FN, thus giving it an important role in ECM maturation and remodeling (Zhang et al., 2014). Despite its relatively sparse expression in adult connective tissues, FN is found abundantly in wound sites (Stoffels et al., 2013). There it enables syndecan-4 and α_5_β_1_-integrin dependent migration of fibroblasts into the provisional matrix (Lin et al., 2005; Miron-Mendoza et al., 2017).

Wound contracture is an important feature throughout the process of wound healing. Accurate matrix contracture reduces the wound area and accounts for over 80% of wound closure *in vivo*, thereby shortening the healing time (Li et al., 2016). Collagen types I and III are the major ECM components of wound granulation tissue (Merkel et al., 1988). As mentioned above, FN is sparse in healthy adult dermis, but is also heavily upregulated in wounds (Stoffels et al., 2013). Fibroblasts, which express collagen-binding receptors as well as FN and α_5_β_1_ integrin, are known to be crucial in mediating collagen matrix contracture in healing wounds (Hinz et al., 2001). Many studies with mesenchymally derived cells have been conducted to establish a function of the major collagen receptors α_2_β_1_ and α_11_β_1_ integrin in collagen gel contracture (Riikonen et al., 1995; Broberg and Heino, 1996; Racine-Samson et al., 1997; Cooke et al., 2000; Barczyk et al., 2013; Liu et al., 2013; Schulz et al., 2015). However, cells of mesenchymal origin also secrete and assemble large amounts of FN in wounds as well as in culture, and in addition, serum FN was present as a component of the culture medium in all *in vitro* models cited above. FN links fibrillar collagen to the cell surface by simultaneously binding to collagen (Erat et al., 2013) and FN-receptor α_5_β_1_ integrin (Pankov and Yamada, 2002). Therefore, it is a reasonable hypothesis that FN in wounds or in collagen gels *in vitro* not only mediates fibroblast adhesion and migration, but could have an important role in collagen matrix contracture itself (Liu et al., 2006). Collagen contracture by activated fibroblasts requires RhoA-mediated actin contractility, and integrin receptors that link the cytoskeleton to the ECM (Hocking et al., 2000; Abe et al., 2007; Clark et al., 2010). In this context, we reported earlier that FN-deficient murine fibroblasts exhibit a defect in mechanotransduction: In the absence of exogenous (serum-derived) FN, FN-null fibroblasts did not respond to tensile strain by RhoA-mediated actin assembly (Lutz et al., 2010). Conversely, FN assembly has been shown to stimulate cell contractility by activating integrin α_5_β_1_, (Hocking et al., 2000). These findings clearly demonstrate that FN–α_5_β_1_ integrin interactions are required for effectively triggering cellular contractility, and support our hypothesis that pericellular FN is involved in collagen matrix contracture.

However, there are conflicting results on this issue in the literature. While one study using inhibitors concluded that fibroblast-mediated collagen gel contracture does not require fibronectin–α_5_β_1_ integrin interactions (Tomasek and Akiyama, 1992), several other groups reported that collagen gel contracture increased, in a concentration-dependent manner, when exogenous FN was added to the culture system (Asaga et al., 1991; Taliana et al., 2000; Nakamura et al., 2003; Liu et al., 2006). Unfortunately, controls including FN-free culture conditions were lacking in these studies.

The aim of the present study was therefore to assess the relative contribution of indirect FN-mediated linkages between cells and fibrillar collagen, vs. direct interactions, to collagen contracture by murine fibroblasts. To address the function of FN in 3D collagen matrix contracture in a direct way, we used immortalized mouse fibroblasts deficient in FN production, which, however, still possess the FN-receptor α_5_β_1_ integrin and thus are able to bind to exogenously added FN. For assessing direct interactions with collagen, we chose two cell lines that differ in their collagen-binding integrins: Embryo-derived FN-null (FN^−/−^) fibroblasts that express α_11_- but essentially no α_2_-integrin subunit, and neonatal kidney-derived fibroblasts that exhibit α_2_- but little α_11_-integrin and in which FN production was suppressed by shRNA transfection. FN-deficient fibroblasts and their wildtype counterparts were cultured in FN-free culture media, seeded on collagen gels, and allowed to contract the collagenous substrate with or without addition of exogenous FN. Our results clearly indicate that although collagen-binding integrins are able to mediate adhesion to and partial contracture of a 3D fibrillar collagen gel by fibroblasts, full activity is achieved only in the presence of cell-assembled FN. This points to an essential role for FN and its receptor α_5_β_1_ integrin in cell contractility and thus 3D collagen matrix contracture by murine fibroblasts *in vitro*.

## Materials and Methods

### Cell Culture

An immortalized mouse embryo fibroblast line with a floxed fibronectin gene (FN^f/f^), and a cell line derived from it in which the fibronectin gene was deleted (FN^−/−^), were used throughout this project. Both cell lines were obtained from R. Fässler, Max Planck Institute for Biochemistry, Martinsried, Germany (Fontana et al., 2005). Stable FN-knockdown fibroblast clones 8.3 and 1.2, isolated from 4 week old mouse kidneys and generated by shRNA transfection, were obtained from our previous work (Lutz et al., 2010). In clone 8.3, FN expression was reduced by 70% compared to the parent cells. Clone 1.2 was transfected with a different shRNA, but silencing of FN was not successful and this cell line was thus used as a control. All cells were maintained on culture dishes (Greiner Bio-One, Huberlab AG, Aesch, Switzerland) in Dulbecco’s modified Eagle’s medium (DMEM; Gibco/Life Technologies, LuBioScience, Lucerne, Switzerland) containing 10% fetal calf serum (FCS; Gibco) and 1% antibiotics/antimycotics (Gibco) at +37°C in a humidified CO_2_-incubator. The medium was changed every 3 days. Upon confluency, cells were passaged by trypsinization (0.05% trypsin-EDTA, Gibco).

### FN Depleted FCS

Fibronectin was separated from FCS by affinity chromatography using a column filled with ∼15 ml gelatin-coupled agarose beads (Sigma-Aldrich, Buchs, Switzerland). Fifty milliliter FCS (Gibco/Life Technologies, LuBioScience, Lucerne, Switzerland) was passed twice over the column and sterile filtered using a 0.2 μm pore filter (VWR International, Radnor, PA, United States). The FN depleted serum was aliquoted and stored at −20°C. The column was washed with 0.5 M urea in PBS (150 mM NaCl, 20 mM Na-phosphate, pH 7.3), and the gelatin-bound FN was eluted with 4 M urea in PBS. The gelatin-agarose column was washed with PBS and stored in BBS (150 mM NaCl, 50 mM Na-borate, pH 8.3) at +4°C. The absence of FN from FN-depleted FCS was checked by immunoblotting (Supplementary Figure S1).

### Purification of Fibronectin

All experiments using exogenous fibronectin were performed with gelatin-affinity purified horse serum fibronectin (Lutz et al., 2010) that was stored in aliquots at −20°C. The biological activity of purified FN was first tested by examining cell adhesion and spreading of FN^−/−^ cells on FN coated, BSA-blocked bacteriological culture dishes before use in other experiments (Supplementary Figure S2).

### Fibrin Gels

To avoid too sudden polymerization, all stock solutions were held on ice. For 4 ml fibrin gel at a final concentration of 10 mg/ml (sufficient for nine wells of a 24-well culture dish), 2 ml sterile filtered fibrinogen solution (20 mg/ml in 0.9% NaCl; Sigma-Aldrich), 100 μl sterile filtered aprotinin (10 mg/ml in ddH_2_O; Sigma-Aldrich) and 40 μl sterile filtered thrombin (Sigma-Aldrich; 200 U/ml in DMEM/F12, Gibco) were carefully mixed in a tube, prior to adding 2 ml of 3% FN-depleted FCS/DMEM. The fibrinogen solution was then immediately transferred to a 24-well dish (400 μl/well), and placed in the incubator allowing the gel to temper at +37°C prior to seeding cells on the top. Since thrombin-induced fibrin polymerization occurs very rapidly, it was unfeasible to prepare more than 4 ml fibrin gel at a time.

### Collagen Gels

Collagen gel cultures were prepared essentially as published before (Trachslin et al., 1999). All stock solutions were stored at +4°C and kept on ice whilst mixing the working gel solution. For 7 ml collagen gel (volume for 16 wells of a 24-well culture dish), we sequentially added to 5.25 ml Collagen R solution (2 mg/ml sterile rat tail collagen type I; SERVA Electrophoresis GmbH, Heidelberg, Germany) the following: 0.7 ml 10× minimum essential medium (MEM; Gibco) containing 5× antibiotics/antimycotics (Gibco), 0.7 ml 10× (0.44 M) NaHCO_3_ (sterile filtered) containing 5× antibiotics/antimycotics, 50 μl 1.5 N NaOH (sterile filtered) and 0.35 ml FN-depleted FCS (diluted to 60% with PBS, resulting in a final concentration of 3% FCS in the gel). The solution was rapidly vortexed. At the end, the generated foam was dissolved by quick run centrifugation at 3000 rpm. To a 24-well culture dish, 400 μl collagen gel solution (final concentration 1.5 mg/ml) was added per well. The dish was incubated for 30 min at 37°C in a humidified CO_2_-incubator to allow gelation of the collagen.

### Cell Spreading on Petri Dishes, Fibrin and Collagen Gels

For analyzing cell spreading on a stiff surface, droplets (40 μl) of 0.5 μM FN were placed on a 10 cm bacteriological petri dish and incubated during 60 min at room temperature. The surface was washed with sterile PBS and the area outside the FN droplets blocked during 15 min with 0.3% BSA in PBS. After washing three times with PBS, 500,000 FN^−/−^ fibroblasts were seeded in serum-free DMEM (Gibco) and placed in the humidified CO_2_-incubator at +37°C. Images were captured after 1, 3, and 24 h incubation using an inverted microscope (Leica DM IL LED) equipped with a 5×/0.12 and a 10×/0.30 NA objective and a digital microscope camera (Leica DFC420C, Twain Version 7.7.1.0). Quantifications were done by counting “round,” “spikey” (cells with at least one protrusion) and “spread” cells on FN on each image as previously described (Ruggiero et al., 2013). To test cell spreading on soft collagen and fibrin matrices, gels were prepared as described above. After solidification of the gels, their surfaces were either left untreated, or coated for 2 h with 100 μl sterile PBS containing 80 μg/ml FN. The supernatant on coated gels was carefully removed with a pipette. 250,000 cells were plated on the gels in 3% FN-depleted FCS/DMEM and placed in the humidified CO_2_-incubator at +37°C. Images were captured and quantified as described above.

### Collagen and Fibrin Gel Contracture

Cured collagen and fibrin gels (see above) were seeded with 250,000 cells per well in 1 ml DMEM containing 3% FN-depleted FCS. After allowing the cells to attach to and populate the 3D matrix for 24 h, gels were carefully detached from the walls of the wells using a thin, sterile spatula. The parameters of the assay (cell density, collagen/fibrin concentration, serum concentration) were adjusted for the FN-expressing control cells to yield approximately 40% matrix contracture 5 h and 50% 24 h after release of the collagen gel from the substrate. Under these conditions, inhibition as well as stimulation of matrix contracture by experimental parameters can be measured in an approximately linear range. Images were captured after 1, 3, 5, and 24 h with the ProgRes CapturePro software by a ProgRes CT3 camera (Jenoptik, Jena, Germany) using an Olympus SZX7 stereo microscope equipped with an ACH 1× objective (7:1 zoom ratio, 0.8× range). Matrix contracture was quantified by subtracting the area defined by the “contracture ring” (which corresponds to the gel border that was initially fixed to the wall of the well) from the initial gel area that covered the entire surface of the well before detachment using ImageJ (Software 1.48q, Rayne Rasband, National Institutes of Health, United States).

### Immunofluorescence Staining

Collagen and fibrin gels that were used for examining matrix contracture ability of FN^f/f^ and FN^−/−^ fibroblasts were subsequently fixed in 4% paraformaldehyde dissolved in PBS, then blocked and cells permeabilized with 3% BSA/0.2% TritonX-100/PBS for 30 min at room temperature. Gels with fixed cells were incubated for 60 min directly in the 24-well dish with either rabbit polyclonal anti-FN antibody (Wehrle-Haller et al., 1991; 1:250 in 3% BSA/0.2% Triton/PBS), rat monoclonal anti-CD49e (α_5_-integrin) antibody (BD Pharmingen; 1:250 in BSA/Triton/PBS), or mouse monoclonal anti-α-smooth muscle (αSM) actin antibody (Sigma-Aldrich; 1:400 in 3% BSA/0.2% Triton/PBS). Samples were washed three times for 7 min with 0.5% BSA/0.03% TritonX-100/PBS. TRITC-phalloidin (1 μg/ml; Sigma-Aldrich) was added along with FITC goat-anti-rabbit IgG (1:250; Sigma-Aldrich) to detect the actin cytoskeleton and the primary antibody against FN, respectively. Alexa Fluor^®^ 568-labeled goat anti-mouse IgG (Invitrogen, Life Technologies, LuBioScience, Lucerne, Switzerland; diluted 1:500 in BSA/Triton/PBS) was added to label the primary antibody against αSM-actin. Alexa Fluor^®^ 488-labeled goat anti-rat IgG (Invitrogen; diluted 1:500 in BSA/Triton/PBS) was used to detect the primary antibody against α_5_-integrin. Each gel culture was washed three times for at least 7 min with BSA/Triton/PBS and carefully transferred from the 24-well dish onto a glass slide using flattened forceps and a fine spatula. Prior to placing a coverslip, the gels were mounted in PBS buffered 90% glycerol containing 1 μg/ml DAPI (Roche, Basel, Switzerland) to stain the nuclei. Images were captured with ProgRes CapturePro software by a ProgRes CT3 camera (Jenoptik, Jena, Germany), using a 40×/0.75 NA objective on an Olympus BX-51 phase/fluorescence microscope equipped with a xenon lamp (X-Cite, series 120PC Q, Mississauga, ON, Canada), and fluorescence filters U-MWIBA3 for Alexa Fluor 488, U-MWIGA3 for Alexa Fluor 568 and TRITC, and U-MNUA2 for DAPI.

### Western Blots

Cell cultures on collagen gels were grown in 3% FN-depleted FCS/DMEM, washed 3× in ice cold sterile PBS and counted. Cells were scratched in RIPA buffer (50 mM Tris-HCl, pH 7.4, 150 mM NaCl, 1% NP-40, 0.25% sodium deoxycholate, and 1 mM EDTA) containing protease and phosphatase inhibitors (Roche, Basel, Switzerland). Debris was removed by centrifugation. Adjusted samples were separated by SDS-PAGE (Laemmli, 1970) and blotted to nitrocellulose membranes. After a blocking step in 1% milk, membranes were incubated with the following antibodies diluted in TBS containing 0.1% Tween and 3% BSA: Rabbit polyclonal anti-fibronectin antibody (1:500, see above), rabbit anti-integrin α_1_ (Santa Cruz Biotechnology, Santa Cruz, CA, United States), anti-integrin α_2_ (1:500) (Zhang et al., 2006) and anti-integrin α_11_ (1:500) (Popova et al., 2007), mouse anti-vinculin (1:500, Sigma-Aldrich) and mouse anti-β-actin (1:400, Sigma-Aldrich). Blots were then incubated for 1 h with either anti-rabbit or anti-mouse IgG coupled to horseradish peroxidase (1:2000; Jackson ImmunoResearch, Suffolk, United Kingdom). Blots were developed using ECL reagent (GE Healthcare, Buckinghamshire, United Kingdom) and scanned by a Storm 840 Phosphoimager (Glattbrugg, Switzerland). Expression levels relative to actin and vinculin expression were calculated by using the measured gray scale values (ImageJ). The blots were repeated twice with the same protein load.

### Cell Proliferation

Proliferation rates were determined using a BrdU labeling reagent (Invitrogen) and a biotin-streptavidin based staining kit (Invitrogen). Cells were incubated for 4 h in sterile-filtered BrdU solution (1:100 in 3% FN-depleted FCS/DMEM), washed 3 × 2 min with PBS, and fixed in 70% ethanol. Endogenous peroxidase was blocked with 3% H_2_O_2_ for 10 min. BrdU positive nuclei were visualized using biotinylated anti-BrdU, streptavidin-peroxidase and diaminobendizine according to the manufacturer’s instructions. Hematoxylin was applied for counterstaining. The cells were mounted in PBS buffered 90% glycerol. Images were captured as described above. To differentiate between the blueish (hematoxylin) and brownish (oxidized DAB) DNA we used a color threshold (ImageJ, see above) in order to quantify the labeled nuclei.

### Inhibition of Fibronectin Receptor α_5_β_1_-Integrin

Trypsinized cells were resuspended in 3% FN-depleted FCS/DMEM, and incubated for 30 min at room temperature with either rat monoclonal anti-CD49e (α_5_-integrin) antibody (10 μg/ml, BD Pharmingen, Basel, Switzerland) (Hocking et al., 2000), or peptides GRGDSP (1 mM) or GRGESP (1 mM, Bachem, Bubendorf, Switzerland). Cell suspensions containing antibody or peptides, respectively, in FN-free FCS/DMEM were then seeded on the collagen gels as described above.

### Statistics

All statistical tests were performed using the software R (version 2.15.1) and Excel 2007 (Windows Corporations, Redmond, Washington, United States). For each experiment, the data were first tested for normality (Shapiro–Wilk test) and homoscedasticity (Levene’s or Bartlett’s test). Based on these results, parametric tests were chosen for examining statistical significance. Depending on the data, either a (unpaired or paired) Student’s *t*-test, one- or two-way ANOVA followed by Tukey’s multiple comparisons test was run (see figure legends for more details). Differences with a value of *p* < 0.05 were considered significant. If not described differently in the text, the graphs indicate the mean ± SD of at least three independent experiments.

## Results

### Integrin Expression by Fibronectin-Deficient and Control Cell Lines

**FIGURE 1:**
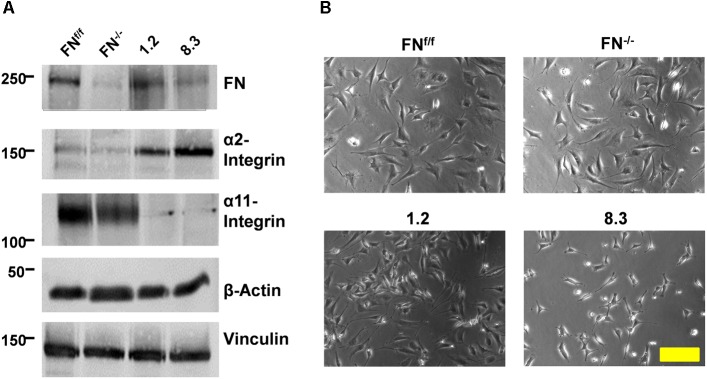
Expression of FN and collagen-binding integrins by FN-deficient vs. control fibroblasts. **(A)** Immunoblots of cell extracts obtained from FN^f/f^, FN^−/−^, clone 1.2, and clone 8.3 fibroblasts. Blots were probed with antibodies to fibronectin (FN), α_2_-integrin, α_11_-integrin, and β-actin and vinculin for loading control. The immunoblot experiments were performed three times; yielding identical results (see Supplementary Figure S3). **(B)** Phase contrast images of FN^f/f^, FN^−/−^, clone 1.2, and clone 8.3 fibroblasts on culture dishes in 10% FCS medium (Scale bar: 100 μm).

Two pairs of FN-deficient mouse fibroblast lines and their respective control cells were used for this study: First, an E13.5 embryonic fibroblast line containing a floxed FN gene (FN^f/f^) and a “null” line with deleted FN gene derived from it (FN^−/−^) (Fontana et al., 2005); and second, two FN-knockdown fibroblast clones, 1.2 and 8.3, derived from postnatal kidney (Graness et al., 2006) and generated by stable shRNA transfection (Lutz et al., 2010). FN^−/−^ cells do not secrete the protein, whereas in clone 8.3, FN expression is reduced by roughly 70% (Figure 1, Supplementary Figure S3). Clone 1.2, transfected with a different shRNA that did not result in FN silencing, expresses normal levels of FN, as do FN^f/f^ cells (Figure 1). In FN-depleted medium, assembly of a pericellular FN meshwork was found in FN^f/f^ and clone 1.2 fibroblasts, and was lacking in cultures of FN^−/−^ and clone 8.3 fibroblasts (Lutz et al., 2010). After addition of exogenous FN, FN fibrils were assembled by all cell lines. We assessed the repertoire of fibronectin- and collagen-binding integrin receptors of the four cell lines by immunoblotting of detergent-solubilized proteins. We showed before (Supplementary Figure S4) that FN receptor subunits α_5_- and β_1_-integrin are equally expressed by FN-null and -knockdown fibroblasts and their respective control lines (Lutz et al., 2010). As shown in Figure 1, all four lines also possess specific collagen-binding integrin receptor subunits (for quantification, see Supplementary Figure S3). Expression levels of α_1_-integrin were very low but similar between all cell lines (Supplementary Figure S5). Intriguingly, however, embryonic FN^−/−^ and FN^f/f^ fibroblasts were found to prominently express α_11_- but little α_2_-integrin, whereas kidney fibroblast clones 8.3 and 1.2 practically lack α_11_- but show high α_2_-integrin expression (Figure 1). The second subunit of these collagen receptors, β_1_-integrin, is constitutively expressed by all four cell lines (Supplementary Figure S4).

### FN-Null and Wildtype Mouse Embryo Fibroblasts Equally Contract a Non-collagenous, Fibronectin-Rich Fibrin Matrix

**FIGURE 2:**
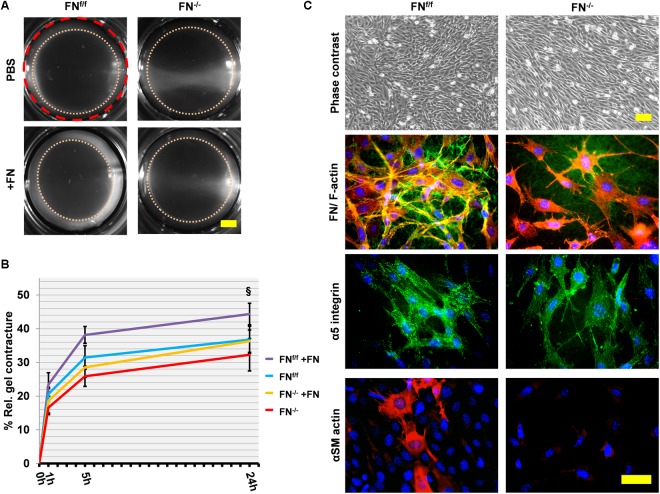
Fibrin contracture and immunofluorescence staining of FN^f/f^ and FN^−/−^ fibroblasts on fibrin 24 h after gel detachment. **(A)** Representative images of fibrin gels 24 h after release. The initial area is given by the dashed red circle. After gel release, a “contracture ring” (pink dotted circle) defined the borders of the measured area with time (Scale bar 2 mm). **(B)** The graph indicates the relative mean fibrin gel contracture of control (FN^f/f^) and fibronectin null (FN^−/−^) fibroblasts on standard (FN-containing) fibrin gels. Data are from at least three independent experiments done in quintuplicates and expressed relative to the initial area (red dashed line) in percent (±SD; significance after 24 h was tested by two-way ANOVA, followed by Tukey’s multiple comparisons test) 0, 1, 5, and 24 h after gel detachment. Overall there was no significant difference between FN^−/−^ and FN^f/f^ fibroblasts on fibrin gels. Adding exogenous FN (20 μg/ml, +FN) slightly but significantly increased the contracture ability of FN^f/f^ cells (^§^*p* < 0.05). **(C)** Immunofluorescence staining of cell cultures on fibrin 24 h after gel detachment: Cells spread and grew easily on fibrin gels as seen in the phase contrast images (top panels; scale bar: 100 μm). FN^f/f^ but not FN^−/−^ fibroblasts assembled a dense FN-network (green) and formed longer fibrillar adhesions containing α_5_ integrin receptors (green). F-Actin (red). Certain FN^f/f^ fibroblasts were positive for αSM-actin (bottom row, Texas Red staining) indicating the existence of myofibroblasts, which were not observed in FN^−/−^ fibroblast cultures (bottom panels; scale bar: 50 μm).

We tested in a preliminary experiment whether FN^−/−^ and FN^f/f^ fibroblasts were able to contract a non-collagenous matrix to a similar extent, independently of their ability to express FN or not. We found that FN^f/f^ as well as FN^−/−^ fibroblasts were perfectly able to adhere and spread on a fibrin matrix that contains FN (Supplementary Figure S6). Next, we detached the fibrin gels from the border of the wells, allowing the cells to contract the fibrin matrix for 24 h (Figure 2A,B). FN^−/−^ and FN^f/f^ fibroblasts exerted statistically indistinguishable fibrin gel contracture. This shows that FN^−/−^ cells possess the machinery to contract a 3D matrix as efficiently as FN^f/f^ fibroblasts. Immunofluorescence staining of fibrin gel cultures revealed subtle FN fibril assembly and α_5_-integrin-positive fibrillar adhesions by FN^−/−^ fibroblasts (Figure 2C). In contrast, FN^f/f^ fibroblasts in fibrin matrix assembled α_5_-integrin in larger and more elongated fibrillar adhesion contacts, which correlated with the presence of a dense FN meshwork around these cells. Actin stress fibers, however, were similarly arranged in both, FN^f/f^ and FN^−/−^ fibroblasts, whereas alpha-smooth muscle-actin (αSM-actin) was expressed in a fraction of FN^f/f^ but barely in FN^−/−^ cells (Figure 2C). In summary, FN^f/f^ and FN^−/−^ fibroblasts were able to exert matrix contracture to an equal extent within 24 h in a non-collagenous, FN-rich environment.

### FN-Null Mouse Embryo Fibroblasts Require Added Fibronectin to Spread on and Contract a Collagen Matrix

**FIGURE 3:**
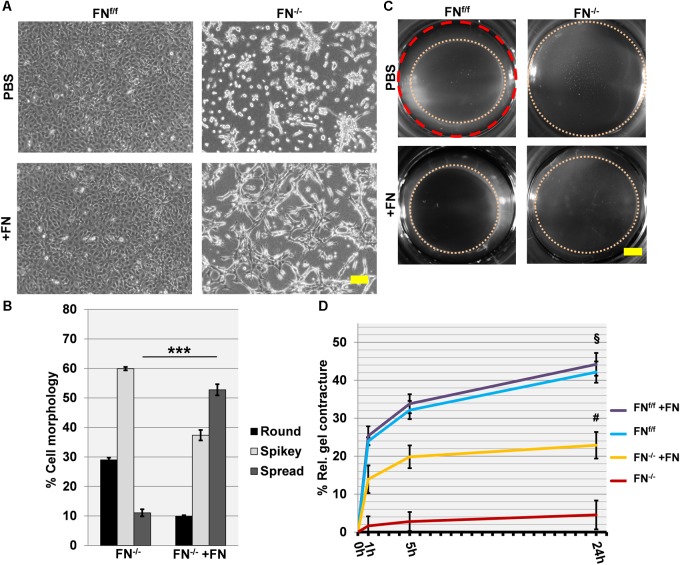
Cell spreading on collagen gels of FN^f/f^ and FN^−/−^ fibroblasts, and their ability to contract a collagen matrix. **(A)** In contrast to wildtype fibroblasts (FN^f/f^, top left panel), fibronectin-null (FN^−/−^) cells did not spread and tended to cluster on collagen gels after 24 h in FN-depleted conditions (top right). Spreading of FN^f/f^ fibroblasts was not influenced by adding 20 μg/ml purified FN to the medium (bottom left). A representative image shows partial rescue of cell spreading by FN^−/−^ cells in the presence of exogenous FN (bottom right) (Scale bar: 100 μm). **(B)** Mean percentage (±SD; significance was tested by a paired *t*-test) of “round” (black bar), “spikey” (light gray bar) and “spread” (dark gray bar) FN^−/−^ fibroblasts on collagen gels 24 h after seeding with or without exogenous FN (^∗∗∗^*p* < 0.001). **(C)** Representative images of collagen gels 24 h after release; the initial area is given by the dashed red circle: After gel release, a “contracture ring” (pink dotted circle) defined the borders of the measured area with time (Scale bar: 2 mm). **(D)** The graph indicates the relative mean gel contracture of FN^f/f^ and FN^−/−^ fibroblasts in the absence or presence of 20 ug/ml FN added to the collagen gel (+FN), 0, 1, 5, and 24 h after gel detachment. Data are from at least three independent experiments done in quintuplicates and are expressed as contracture relative to the initial area in percent (±SD; significance after 24 h was tested by two-way ANOVA, followed by Tukey’s multiple comparisons test). FN^f/f^ fibroblasts exerted roughly a nine-fold higher collagen contracture ability after 24 h compared to FN^−/−^ cells (^§^ *p* < 0.001). Addition of exogenous FN did not affect control fibroblasts. Collagen contracture exerted by FN^−/−^ cells was partially rescued by adding exogenous FN to the collagen gels, which caused a five-fold increase (^#^*p* < 0.001).

We then proceeded to seed cells on a collagen matrix with or without exogenous FN (∼20 μg per ml of collagen gel). Unlike on fibrin, FN^−/−^ fibroblasts attached to a native collagen gel but had major difficulties to spread under FN-free conditions. They appeared roundish and tended to form clusters (Figure 3A). However, the addition of 20 μg/ml exogenous FN significantly increased the amount of spread cells after 24 h of incubation (Figure 3A,B; *p* < 0.001). The rescue was partial, since a higher FN concentration did not increase cell spreading (Supplementary Figure S7). As expected, FN^f/f^ cells fully spread on collagen gels, and spreading remained unchanged after adding supplemental FN. Note that both FN^f/f^ and FN^−/−^ fibroblasts express α_11_- but almost no α_2_-integrin as specific collagen receptor (Figure 1). Our results therefore indicate that α_11_β_1_-integrin does not mediate efficient spreading of mouse embryo fibroblasts on a soft collagen matrix, but that cellular FN (via its receptor α_5_β_1_-integrin) can compensate for the lack of a spreading-promoting collagen receptor.

After 24 h, collagen gels with plated cells were released from the culture well to initiate contraction (Figure 3C,D), and matrix contracture was quantified at different time points after release. Clearly, FN^−/−^ fibroblasts in FN-depleted medium were seriously compromised in their ability to contract the collagen matrix. The relative contracture after 24 h was roughly nine-fold below the one exerted by FN^f/f^ cells (*p* < 0.001). Differences in cell proliferation rates that could influence collagen gel contracture were not observed between both cell lines (Supplementary Figure S8). A five-fold increase in contracture was obtained for FN^−/−^ fibroblast cultures after adding exogenous FN to the collagen matrix (*p* < 0.001), but a full rescue to the level of FN^f/f^ cells was not achieved (Figure 3C,D). As expected, no increase was recorded for FN^f/f^ cells after FN addition.

**FIGURE 4:**
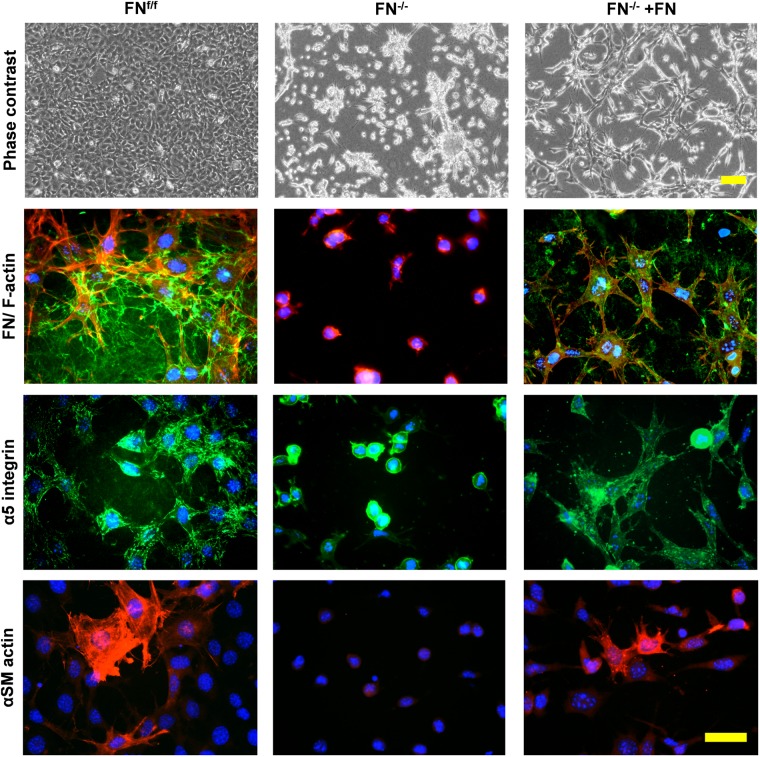
Immunofluorescence staining of FN^f/f^ and FN^−/−^ fibroblasts on contracted collagen gels. Control (FN^f/f^) fibroblasts spread extensively on native collagen, whereas FN-null (FN^−/−^) cells appeared roundish and tended to form clusters. In the presence of exogenous FN, FN^−/−^ fibroblasts increasingly spread as seen on the phase contrast images (top panels; scale bar 100 μm). The assembly of a FN network (green) and α_5_ integrin in focal adhesions (green) by FN^−/−^ cells in the presence of exogenous FN could also be observed (middle panels; F-actin in red). Mature myofibroblasts were present amongst FN^f/f^ fibroblasts; these were characterized by a well-organized alpha-smooth muscle actin (αSM-actin) positive cytoskeleton (bottom panels; Texas Red staining). A fraction of FN^−/−^ fibroblasts showed higher αSM-actin expression in the presence of exogenous FN (bottom panels; scale bar 50 μm).

Cells were then stained for FN, α_5_-integrin and αSM-actin (Figure 4). A dense FN meshwork around FN^f/f^ fibroblasts was visible, in contrast to what was observed for FN^−/−^ cultures under FN-depleted conditions. Long α_5_-integrin positive fibrillar adhesions were regularly distributed over the FN^f/f^ cell surface, and myofibroblasts appeared scattered as very large cells in pairs or small islets with αSM actin-positive bundles. In obvious contrast, FN^−/−^ cells in collagen gels under FN-depleted conditions appeared roundish and lacked fibrillar adhesions, organized actin stress fibers, as well as αSM-actin expression. However, a dramatic change was observed after addition of exogenous FN. Instead of resting on the gel surface, FN^−/−^ fibroblasts formed cell processes in all dimensions (see F-actin, Figure 4). In addition, a thin assembled FN network was identifiable, which correlated with α_5_-integrin positive fibrillar adhesion contacts all over the cell surface. Moreover, a very small fraction of cells (23–30 cells per gel) showed elevated αSM actin expression.

### FN-Knockdown Fibroblasts From Postnatal Mice Exhibit Impaired Collagen Gel Contracture to a Lesser Extent

**FIGURE 5:**
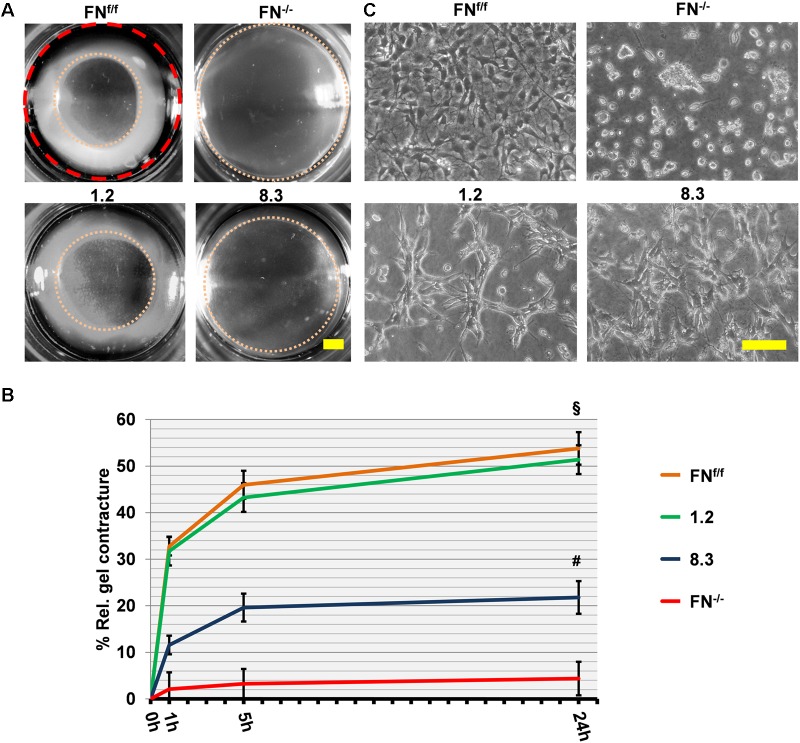
Collagen gel contracture by FN-knockdown fibroblast clones 1.2 and 8.3. **(A)** Representative images of collagen gel contracture are shown as described above (Scale bar: 2 mm). **(B)** The graph indicates the mean gel contracture relative to the initial area (red dashed circle) in percent by fibroblast clones 1.2 (normal FN expression) and 8.3 (70% reduced FN expression), compared to control (FN^f/f^) and fibronectin-null (FN^−/−^) fibroblasts. Data are from at least three independent experiments (±SD; significance after 24 h was tested by two-way ANOVA, followed by Tukey’s multiple comparisons test), 0, 1, 5, and 24 h after gel detachment. Clone 1.2 showed similar collagen contracture ability as FN^f/f^ fibroblasts, whereas clone 8.3 exerted a contracture that was substantially reduced compared to the control cells (^§^ *p* < 0.001), but higher than that of FN^−/−^ cells (^#^*p* < 0.001). **(C)** Phase contrast images of fibroblasts on collagen gels 24 h after release: Cells from clone 1.2 did not spread to the same extent as FN^f/f^ fibroblasts on native collagen (Scale bar: 100 μm).

To confirm our findings, we also examined postnatal fibroblasts in which FN expression was suppressed by shRNA-mediated knockdown. By direct comparison, FN-expressing clone 1.2 and FN^f/f^ fibroblasts contracted collagen gels to an equal extent in FN-free medium (Figure 5). Like FN^−/−^ fibroblasts, FN-deficient clone 8.3 showed impaired collagen gel contracture compared to the control lines, clone 1.2 and FN^f/f^ fibroblasts, respectively (Figure 5; *p* < 0.001). However, matrix contracture of clone 8.3 after 24 h was higher relative to that exerted by FN^−/−^ fibroblasts (*p* < 0.001). This might be partially due to the fact that clone 8.3 showed enhanced spreading behavior compared to FN^−/−^ fibroblasts (Figure 5), and in fact spread in a similar manner as clone 1.2 on collagen gel. Note that clone 8.3 secretes a small amount of residual FN, and that both clones 1.2 and 8.3 express α_2_β_1_-integrin (Figure 1), which might explain the differences to FN^f/f^ and FN^−/−^ fibroblasts, respectively. Nevertheless, FN-deficient clone 8.3 did by far not reach the same level of collagen gel contracture as control clone 1.2 in a FN-depleted environment.

**FIGURE 6:**
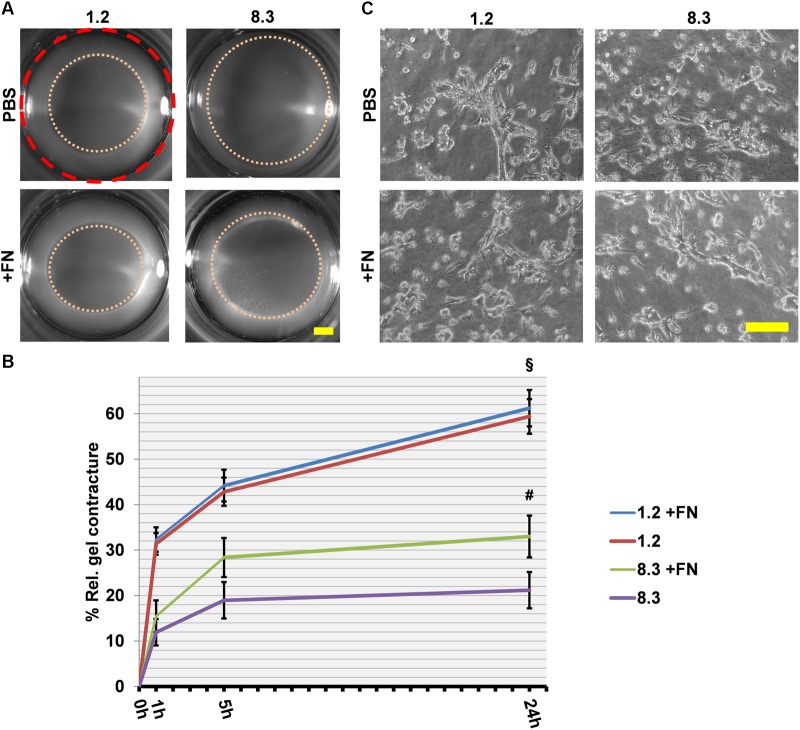
Collagen gel contracture by FN knockdown fibroblast clones 1.2 and 8.3 with or without exogenous FN. **(A)** Representative images of gel cultures are shown as described above (Scale bar: 2 mm). **(B)** The graph indicates the relative mean gel contracture in relation to the initial area (red dashed circle) in percent from at least three independent experiments (±SD significance after 24 h was tested by two-way ANOVA, followed by Tukey’s multiple comparisons test) 0, 1, 5, and 24 h after gel detachment. The collagen gel contracture exerted by FN-deficient clone 8.3 supplemented with exogenous FN is statistically greater than that by clone 8.3 in FN-depleted conditions (^#^*p* < 0.001). Clone 1.2 contracted collagen gels more extensively than did clone 8.3 with or without exogenous FN (^§^ *p* < 0.001). **(C)** Phase contrast images of fibroblast clones 1.2 and 8.3 on collagen gels 24 h after detachment: No differences can be seen in cell spreading with or without exogenous FN (Scale bar 100 μm).

We further investigated the importance of cell-assembled FN in collagen matrix contracture, by performing a rescue assay using clones 8.3 and 1.2 with exogenous FN (Figure 6), as done before with FN^−/−^ and FN^f/f^ fibroblasts. A significant increase in collagen gel contracture was observed for clone 8.3 but not clone 1.2 after addition of FN (*p* < 0.001). However, cell spreading remained unchanged for both clones in the presence of exogenous FN (Figure 6). In summary, FN-knockdown cells from clone 8.3 exhibited higher residual collagen contracture than FN-null (FN^−/−^) fibroblasts. Nevertheless, both cell lines deficient in FN expression had a reduced ability to contract a collagen matrix, and required exogenous FN in order to increase this activity.

### Blocking α_5_-Integrin Function Attenuates Collagen Gel Contracture

**FIGURE 7:**
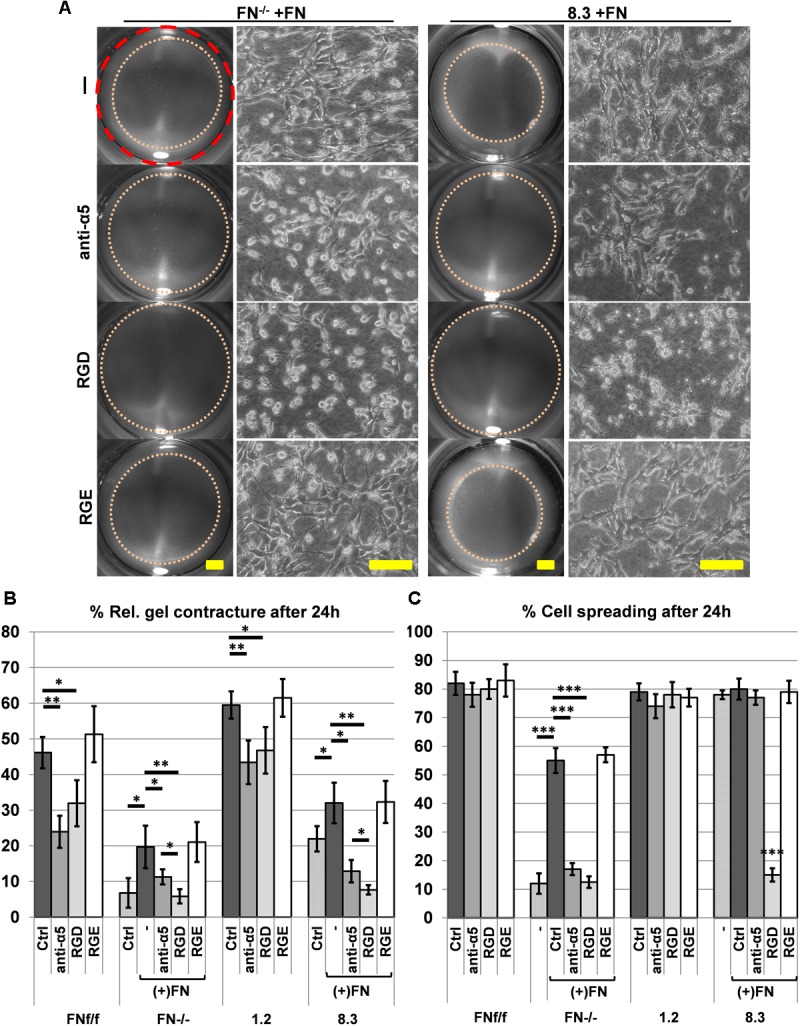
Effect of blocking integrin α_5_β_1_ function on collagen gel contracture and cell spreading by normal and FN-deficient fibroblasts. Cells were cultured on collagen gels in 3% FN-depleted FCS/DMEM without (Ctrl: control) or with 20 μg/ml exogenous FN (+FN). Cultures were either left untreated (–), or alternatively a function blocking antibody against integrin α_5_ (anti-α_5_, 10 μg/ml), adhesion-blocking peptide GRGDSP (RGD, 1 mM), or control peptide GRGESP (RGE, 1 mM) were added to the medium. **(A)** Representative images of contracted collagen gels (Scale bar: 2 mm) and the respective spreading behavior of FN-deficient (FN^−/−^, clone 8.3) cell lines after 24 h in the presence of exogenous FN without (-; RGE) or with (anti-α_5_, RGD) inhibitors added (Scale bar: 100 μm). Note that both anti-α_5_ integrin antibody and GRGDSP peptide reduce FN-mediated cell spreading and matrix contracture on collagen gel. **(B)** Mean gel contracture relative to the initial area in percent from three independent experiments (±SD significance after 24 h was tested by two-way ANOVA, followed by Tukey’s multiple comparisons test) done in triplicates 24 h after gel detachment. Both, anti-α_5_ integrin antibody (anti-α_5_) and peptide GRGDSP (RGD) significantly reduced collagen gel contracture by control fibroblasts (FN^f/f^, clone 1.2) in FN-depleted medium, and completely inhibited contracture ability of FN-deficient cells (FN^−/−^, clone 8.3) stimulated by exogenous FN (^∗^*p* < 0.05; ^∗∗^*p* < 0.01). **(C)** Mean rate of spread cells relative to the total amount of cells in percent from three independent experiments (±SD; significance after 24 h was tested by two-way ANOVA, followed by Tukey’s multiple comparisons test) after 24 h of incubation on collagen gels. Cell spreading of FN^−/−^ fibroblasts was significantly reduced when anti-α_5_ integrin antibody (anti-α_5_) and GRGDSP (RGD) where added to the culture media. 8.3 showed reduced spreading when incubated with the RDG peptide but not with the anti-α_5_ integrin antibody (^∗∗∗^*p* < 0.001).

Since α_5_β_1_-integrin is the major FN receptor of fibroblasts, FN-dependent collagen gel contracture should be inhibited by blocking its binding to FN. To test this notion, FN-deficient fibroblasts FN^−/−^ and 8.3 were supplemented with exogenous FN that allowed them to contract the collagen matrix. This FN-induced contracture was effectively abolished in the presence of a blocking anti-α_5_ integrin antibody or the peptide GRGDSP, which mimics the integrin binding site on FN (Figure 7A). In comparison to the specific anti-α_5_ integrin antibody, GRGDSP was even more potent in reducing collagen gel contracture by FN-deficient fibroblasts (Figure 7B, *p* < 0.01). GRGDSP also affected cell spreading of 8.3 fibroblasts on collagen gel (Figure 7A, C, *p* < 0.001). Likewise, spreading of FN^−/−^ fibroblasts was reduced on collagen gel in the presence of GRGDSP as well as anti-α_5_ integrin antibody (Figure 7A,C, *p* < 0.001). In contrast, FN-producing FN^f/f^ and 1.2 fibroblasts appeared to spread normally on collagen gels in the presence of either blocking reagent (Figure 7C, Supplementary Figure S9). Nevertheless, collagen gel contracture was significantly reduced when FN-expressing fibroblast lines were incubated with anti-α_5_ integrin antibody or GRGDSP, respectively, in FN-depleted medium (Figure 7B, Supplementary Figure S9). Although collagen gel contracture by control cells was not fully abolished in the presence of either inhibitor, these results confirm an involvement of cell-derived FN and its receptor in efficient collagen matrix contracture by murine fibroblasts.

## Discussion

Contracture of the extracellular matrix, an important feature of wound healing, is achieved by RhoA-dependent actin contractility, and requires integrin receptors that link the ECM to the cytoskeleton (Hocking et al., 2000; Abe et al., 2007; Clark et al., 2010). Collagen types I and III as well as fibronectin are the major ECM components of wound granulation tissue (Merkel et al., 1988). For the major collagen-specific integrins α_2_β_1_ and α_11_β_1_, a role in collagen matrix contracture has been reported (Riikonen et al., 1995; Broberg and Heino, 1996; Cooke et al., 2000; Tiger et al., 2001; Barczyk et al., 2013; Schulz et al., 2015). However, cells of mesenchymal origin also express substantial amounts of FN in wounds and in culture, and FN links collagen to the cell surface by simultaneously binding collagen and FN-receptor α_5_β_1_ integrin. Therefore, FN could also have an important role in wound contracture. As outlined in the Introduction, published results on this issue are contradictory (Asaga et al., 1991; Tomasek and Akiyama, 1992; Taliana et al., 2000; Nakamura et al., 2003; Liu et al., 2006). However, controls including FN-free culture conditions were lacking in these studies. With the present work, we therefore aimed to examine the impact of FN on collagen gel contracture by using FN-deficient fibroblasts in FN-depleted medium. FN-null cells were able to contract a 3D non-collagenous fibrin matrix, showing that their contractile apparatus is functional. On the other hand, our findings demonstrate that effective collagen gel contracture by FN-deficient fibroblasts critically depends on the presence of exogenous FN. This is in accordance with work from another group, which examined the assembly of exogenously added FN and its stimulation of collagen gel contracture by FN-null fibroblasts (Hocking et al., 2000). However, this earlier study did not distinguish between the relative contributions of FN-dependent and -independent mechanisms in collagen gel contracture by FN-null cells, which was the second aim of the present paper.

### Effective Collagen Gel Matrix Contracture Depends on FN and Its Receptor Integrin α_5_β_1_ Independently of α_2_- or α_11_-Integrin Expression

Despite of the expression of major collagen-binding integrins by all fibroblast lines, FN-deficient fibroblasts showed reduced collagen matrix contracture compared to their respective control cells. Embryonic FN^−/−^ fibroblasts, which primarily expressed α_11_- but little α_2_-integrin as major collagen receptor, strictly required exogenous FN to allow the initiation of collagen matrix contracture. This correlated with the formation of dendritic-like protrusions and α_5_β_1_-integrin-positive focal adhesions decorated by freshly assembled FN fibrils in the surrounding collagen matrix. Collagen contracture by FN^−/−^ fibroblasts stimulated by exogenous FN was completely abolished by blocking integrin α_5_β_1_ function. Remarkably wildtype fibroblasts, and a small fraction of FN^−/−^ fibroblasts after addition of exogenous FN, expressed αSM-actin in collagen gels. The role of FN in the appearance of myofibroblasts, and their possible contribution to collagen gel contracture (Tomasek et al., 2013) was not further investigated.

In contrast to FN-null cells, FN-knockdown fibroblasts derived from postnatal mouse kidney expressed α_2_-integrin but lacked the α_11_ subunit. Integrin α_2_β_1_ is a specific collagen receptor that was reported to contribute to fibroblast spreading on soft collagen matrix, as well as collagen contracture (Riikonen et al., 1995; Broberg and Heino, 1996; Racine-Samson et al., 1997; Cooke et al., 2000). Postnatal FN-knockdown fibroblasts (clone 8.3) contracted the collagen matrix to some extent even under FN-free conditions, which might be due to residual FN expression or direct collagen–integrin α_2_β_1_ interactions or both. In any case, contracture by FN-knockdown fibroblasts was significantly enhanced after adding exogenous FN to the collagen gel, and again this increase was effectively suppressed by inhibiting integrin α_5_β_1_. Moreover, anti-α_5_ antibody or GRGDSP peptide (which blocks other RGD-dependent integrins but not the specific collagen receptors α_2_β_1_ and α_11_β_1_) at least partially inhibited collagen matrix contracture by FN-expressing control fibroblasts. The combined data indicate that FN-bound α_5_β_1_ integrin can act in cooperation with either α_11_β_1_ or α_2_β_1_ integrin, but that especially for embryonic fibroblasts lacking α_2_β_1_ integrin, FN is an essential (and sufficient) mediator of collagen gel contracture.

Besides integrin α_5_β_1_, the most prominent alternative FN receptors on fibroblasts are the (promiscuous) RGD-binding integrins α_v_β_1_ and α_v_β_3_, and we have shown previously that all our four cell lines express the α_v_ chain (Lutz et al., 2010) (Supplementary Figure S4). Our experiments revealed that peptide GRGDSP (which interferes with binding of both α_5_β_1_ and α_v_ integrins to FN) was slightly more efficient than anti-α_5_ antibody in blocking collagen contracture by FN-deficient cells after adding exogenous FN. On the other hand, no difference was found between peptide and antibody in inhibiting collagen contracture by FN-expressing cells. Thus, although α_v_-integrins might contribute to FN-dependent collagen contracture to some extent, specific FN receptor α_5_β_1_ is responsible for most of the activity.

Interestingly, the group of D. Gullberg tested the collagen matrix contracture ability of C2C12 myoblasts that do not express any collagen-binding integrins (Tiger et al., 2001), but do produce FN and α_5_β_1_ integrin (Neveux et al., 2010). It is known that myoblasts need FN to spread on gelatin (Chiquet et al., 1979). Thus, C2C12 myoblasts provide an ideal control to test the effects of collagen-bound FN and its receptor α_5_β_1_ integrin in total absence of collagen-binding integrins. Indeed, C2C12 myoblasts were able to contract collagen gels to 60% of their initial gel area in starved culture conditions (Tiger et al., 2001). C2C12 myoblasts transfected with either α_2_- or α_11_-integrin exerted an increased collagen gel contracture, in both cases reducing the gel area to roughly 15% of the initial area. Whereas these published data imply that both collagen-binding receptors can function additively or synergistically with FN-activated integrin α_5_β_1_ in collagen matrix contracture, our present results point to distinct roles of integrins α_2_β_1_ vs. α_11_β_1_ in this process.

### Contribution of Alternative Collagen Receptors to Collagen Matrix Contracture

There remains the question which role other collagen receptors play in collagen matrix contracture in our model. We focused on α_2_β_1_ and α_11_β_1_ integrins because they are reported to be the two major and specific receptors for fibrillar collagens (Tiger et al., 2001; Tulla et al., 2001), and because for both a function in collagen contracture has been reported for specific cell types (Broberg and Heino, 1996; Cooke et al., 2000; Barczyk et al., 2013). Of the other collagen-binding integrins, α_1_β_1_ is known to have higher affinity for non-fibrillar collagens (e.g., basement membrane collagen IV) (Tulla et al., 2001), and moreover it is not specific for collagens as it also recognizes laminin (Calderwood et al., 1997). On immunoblots, we found little or no expression of α_1_-integrin by our four cell lines (Supplementary Figure S5). The fourth integrin that contains a collagen-binding I-domain, α_10_β_1_, is primarily expressed on chondrocytes rather than fibroblasts (Camper et al., 1998), and its ligand specificity has not been studied extensively yet. A recent paper claims that all integrins with a collagen-binding I-domain in fact bind to monomeric collagen rather than fibrils *in vivo*, and suggests that their function in collagen matrix contracture needs to be reconsidered (Woltersdorf et al., 2017). On the other hand, the collagen-binding discoidin domain receptors (DDR) are receptor tyrosine kinases. Although they reportedly mediate adhesion to collagens, their main function appears to be in signal transduction, primarily by inducing MMP production for collagen matrix remodeling (Itoh, 2018). Since their intracellular domain is not known to connect to the actin cytoskeleton (unlike that of integrins), their mechanical function is questionable, and there is no evidence for an involvement in collagen contracture. In our experiments, FN null (FN^−/−^) fibroblasts completely failed to spread on and contract a fibrillar collagen gel; besides FN, these cells lack α_2_-integrin. Conversely, FN-deficient fibroblast clone 8.3, which does express α_2_-integrin, exhibited some degree of collagen contracture, which was increased by adding exogenous FN. This indicates that at least in our fibroblasts, FN-binding α_5_β_1_ and collagen-binding α_2_β_1_ are the most important cellular force transmitters in collagen matrix contraction, and contribution by other collagen receptors must be marginal.

### Cell Spreading Does Not Strictly Correlate With Collagen Gel Contracture

FN^f/f^ fibroblasts spread very easily on collagen gels. This was evidenced by the formation of dendritic-like protrusions invading the collagen matrix. In contrast and most strikingly, FN^−/−^ fibroblasts appeared roundish and tended to form clusters. Exogenous FN reversed cluster formation of FN^−/−^ fibroblasts and initiated the extension of cellular protrusions. A full rescue, however, was not achieved indicating that other factors influence cell spreading. FN^f/f^ and FN^−/−^ fibroblasts express α_11_- but not α_2_-integrin, which, depending on the availability of FN, could affect cell spreading on collagen as well. The role of α_2_-integrin in cell spreading on collagen has been described (Jokinen et al., 2004), but not the one of α_11_-integrin. Surprisingly, there is no study reporting an interaction of collagen-binding receptors and FN-activated integrins in general.

A less obvious difference in cell spreading on collagen was seen when comparing FN-expressing fibroblast line 1.2 with FN-knockdown line 8.3, independently of their ability to contract collagen gels. For line 8.3, spreading was not influenced by the addition of exogenous FN to the collagen gels. The knockdown fibroblast lines (1.2 and 8.3) expressed α_2_-integrin but lacked α_11_-integrin. From our combined results, it appears that at least in our cells, integrin α_2_β_1_ but not α_11_β_1_ suffices to induce fibroblast spreading on soft collagen gels, whereas neither of the two major collagen receptors can mediate efficient collagen gel contracture on its own in the absence of FN. It can therefore be summarized that collagen matrix contracture strongly depends on FN–α_5_β_1_ integrin interactions, but does not strictly correlate with the ability of fibroblasts to spread on collagen gels, which is influenced by the repertoire of collagen receptors.

## Conclusion

Contracture of fibrillar collagen gels by cultured fibroblasts *in vitro* has long been used as a paradigm for wound contraction *in vivo*, but we are well aware of the limitations of this model: The 3D matrix is reduced to pure collagen I and much softer than in real wounds, and instead of primary dermal fibroblasts, we employed genetically modified murine cell lines for our experiments. Nevertheless, we believe that such a reductionist approach is valid and useful for dissecting the function of a single cell-derived ECM component, such as FN, in this process. Within the limits of our *in vitro* study, we demonstrated that FN-deficient fibroblasts require exogenous FN to effectively contract a 3D collagen gel matrix, independently of the expression of major collagen-binding integrins. Contracture is linked to the assembly of FN fibrils and α_5_-integrin-positive fibrillar adhesion contacts. Furthermore, our findings indicate that FN-bound integrin α_5_β_1_ and collagen-binding integrins (primarily α_2_β_1_) appear to act additively to mediate cell spreading and collagen gel contracture. To our knowledge, there is no report that examined the possibility of a cooperation between the respective receptors in activating RhoA-mediated actin contractility in fibroblasts, and this remains to be investigated in a future study. In view of the large secretion of FN by activated fibroblasts in wounds, our present results strongly support the notion that this ECM protein plays a major role in collagen matrix contracture *in vivo* as well.

## Data Availability

All datasets generated for this study are included in the manuscript and/or the Supplementary Files.

## Author Contributions

JB performed the experiments, analyzed the data and wrote the manuscript, CK critically revised the manuscript and provided support throughout the project, MC coordinated and designed the experiments and contributed in writing the manuscript.

## Conflict of Interest Statement

The authors declare that the research was conducted in the absence of any commercial or financial relationships that could be construed as a potential conflict of interest.

## Supplementary Material

FIGURE S1 Fibronectin (FN) was successfully removed from FCS by affinity chromatography. Immunoblots with normal FCS and FN-depleted FCS were probed with a polyclonal antibody to FN. FN was almost undetectable in FN-depleted FCS.

FIGURE S2 Spreading of FN^−/−^ fibroblasts on FN-coated and BSA-blocked bacteriological petri dishes. **(A)** The graph indicates the ratio in percentage (±SD) of “round” (black bars), “spikey” (light gray bars) and “spread” (dark gray bars) cells on FN-coated and BSA-blocked plastic, respectively. **(B)** Representative images after 24 h of incubation. On BSA-blocked dishes, FN^−/−^ fibroblasts did not adhere and seemingly died after 24 h. Complete spreading was achieved on FN-coated plastic, indicating that FN^−/−^ cells possess functional FN receptors (^∗∗∗^*p* < 0.001, paired *t*-test) (Scale bar: 50 μm).

FIGURE S3 Quantification of FN and integrins from immunoblots. **(A)** Another example of an immunoblot as shown in Figure 1A, but from a replicate experiment, with cell extracts obtained from FN^f/f^, FN^−/−^, clone 1.2, and clone 8.3 fibroblasts. Blots were probed as described with antibodies to fibronectin (FN), α_2_-integrin, α_11_-integrin, and β-actin and vinculin for loading control. **(B)** The values indicate the mean protein expression (±SD) of FN, α_2_- and α_11_-integrin normalized to β-actin (top graphs) or vinculin (bottom graphs) from three independent experiments (^∗^*p* < 0.05; ^∗∗^*p* < 0.01; one-way ANOVA, followed by Tukey’s multiple comparisons test).

FIGURE S4 Similar expression of integrin chains α_v_, α_5_, and β_1_ by FN-deficient vs. control fibroblasts. Immunoblots of cell extracts obtained from FN^f/f^, FN^−/−^, clone 1.2, and clone 8.3 fibroblasts. Blots were probed with antibodies to the respective integrin chains, and GAPDH for loading control. Modified from Lutz et al. (2010).

FIGURE S5 Expression level of integrin**-**α_1_ is very low but similar between all cell lines. Immunoblot of cell extracts obtained from FN^f/f^, FN^−/−^, clone 1.2, and clone 8.3 cells. Blots were probed with antibodies to integrin-α_1_, and with β-actin and vinculin for loading control.

FIGURE S6 Spreading of FN^f/f^ and FN^−/−^ fibroblasts on FN-containing fibrin gels 3 h after seeding. **(A)** Representative images taken 30 min and 3 h after seeding cells (Scale bar: 100 μm). **(B)** The graph indicates the ratio in percentage (±SD) of “round” (black bars), “spikey” (light gray bars) and “spread” (dark gray bars) cells relative to the total number of cells. Statistical evaluation includes the average percentage of spread cells from three independent measurements (^∗^*p* < 0.05, unpaired *t*-test).

FIGURE S7 Doubling exogenous FN concentration does not further increase cell spreading of FN^−/−^ fibroblasts on a 3D collagen matrix. **(A)** FN^−/−^ cells require addition of 20 μg/ml exogenous FN to partially spread on collagen gel. Higher FN concentration (40 μg/ml) did not further enhance this behavior (Scale bar: 100 μm). **(B)** The ratio of spread FN^−/−^ cells is statistically not significantly different (n.s.) between the two concentrations (20 or 40 μg/ml) of exogenous FN (±SD of three independent measurements, paired *t*-test).

FIGURE S8 Similar proliferation rates of FN-deficient and control fibroblasts. **(A)** Representative images of BrdU-labeled cells, revealing newly synthesized DNA (brown) in a fraction of them. Hematoxylin (blue) was used as counterstaining. **(B)** Mean ratio of BrdU-positive cells relative to unstained (blue) cells as a percentage (±SD of three independent measurements, unpaired *t*-test) showed no difference between FN-deficient and control fibroblasts (Scale bar: 200 μm).

FIGURE S9 Impeded collagen gel contracture by FN^f/f^ and 1.2 fibroblasts after adding function-blocking anti-integrin α_5_ antibody or RGD peptide. Representative images of contracted collagen gels (left panels; scale bar: 2 mm) and the respective spreading behavior FN^f/f^ and 1.2 fibroblasts after 24 h (right panels; scale bar: 100 μm). Cultures were either left untreated (−), or alternatively a function blocking antibody against integrin α_5_ (anti-α_5_, 10 μg/ml), adhesion-blocking peptide GRGDSP (RGD, 1 mM), or control peptide GRGESP (RGE, 1 mM) were added to the medium. Collagen gel contraction was impeded after blocking integrin α_5_ by the respective antibody or by GRGDSP. Cell spreading was not affected by either inhibitor (see Figure 7 for quantification).
