# Supplementary figures and images for: Impaired Contracture of 3D Collagen Constructs by Fibronectin-Deficient Murine Fibroblasts
Source: Front Physiol. 2019 Mar 5;10:166. doi: 10.3389/fphys.2019.00166 (PMC6413635; doi:10.3389/fphys.2019.00166)

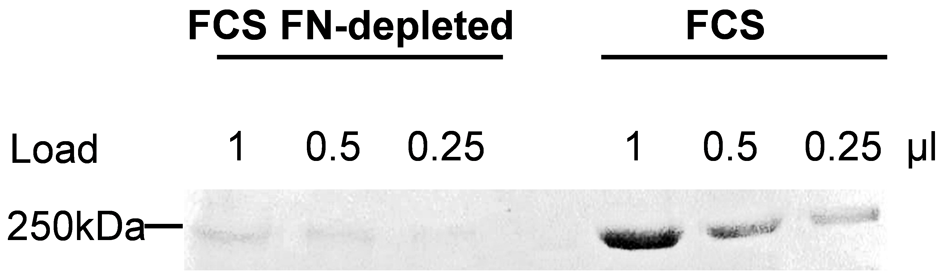

Supplement: FIGURE S1 — Fibronectin (FN) was successfully removed from FCS by affinity chromatography. Immunoblots with normal FCS and FN-depleted FCS were probed with a polyclonal antibody to FN. FN was almost undetectable in FN-depleted FCS. [file Image_1.TIF]

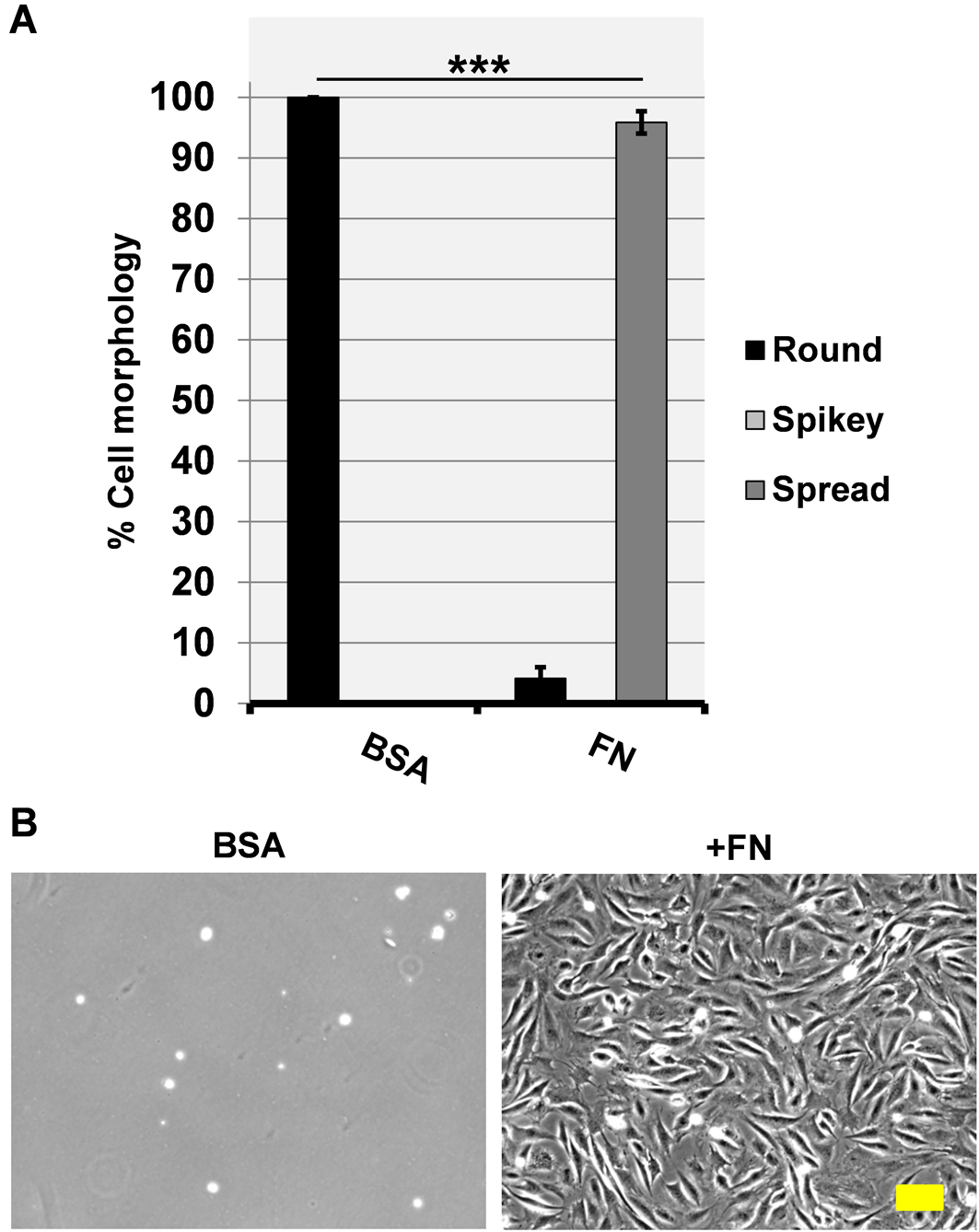

Supplement: FIGURE S2 — Spreading of FN−/− fibroblasts on FN-coated and BSA-blocked bacteriological petri dishes. (A) The graph indicates the ratio in percentage (±SD) of “round” (black bars), “spikey” (light gray bars) and “spread” (dark gray bars) cells on FN-coated and BSA-blocked plastic, respectively. (B) Representative images after 24 h of incubation. On BSA-blocked dishes, FN−/− fibroblasts did not adhere and seemingly died after 24 h. Complete spreading was achieved on FN-coated plastic, indicating that FN−/− cells possess functional FN receptors (∗∗∗p < 0.001, paired t-test) (Scale bar: 50 μm). [file Image_2.TIF]

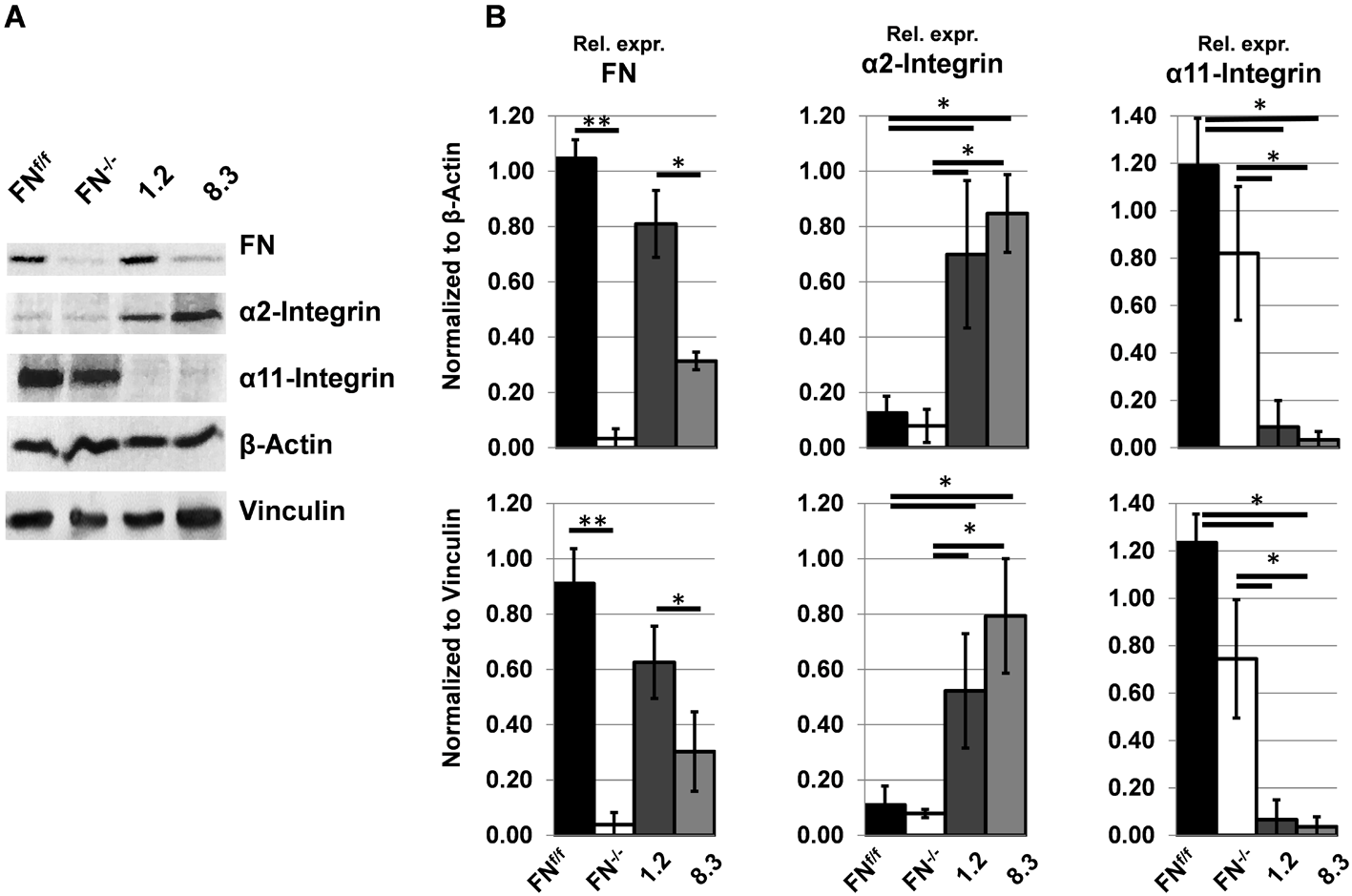

Supplement: FIGURE S3 — Quantification of FN and integrins from immunoblots. (A) Another example of an immunoblot as shown in Figure 1A, but from a replicate experiment, with cell extracts obtained from FNf/f, FN−/−, clone 1.2, and clone 8.3 fibroblasts. Blots were probed as described with antibodies to fibronectin (FN), α2-integrin, α11-integrin, and β-actin and vinculin for loading control. (B) The values indicate the mean protein expression (±SD) of FN, α2- and α11-integrin normalized to β-actin (top graphs) or vinculin (bottom graphs) from three independent experiments (∗p < 0.05; ∗∗p < 0.01; one-way ANOVA, followed by Tukey’s multiple comparisons test). [file Image_3.TIF]

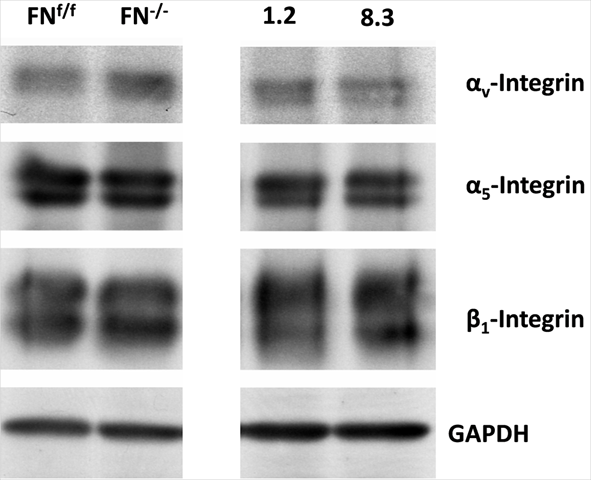

Supplement: FIGURE S4 — Similar expression of integrin chains αv, α5, and β1 by FN-deficient vs. control fibroblasts. Immunoblots of cell extracts obtained from FNf/f, FN−/−, clone 1.2, and clone 8.3 fibroblasts. Blots were probed with antibodies to the respective integrin chains, and GAPDH for loading control. Modified from Lutz et al. (2010). [file Image_4.TIF]

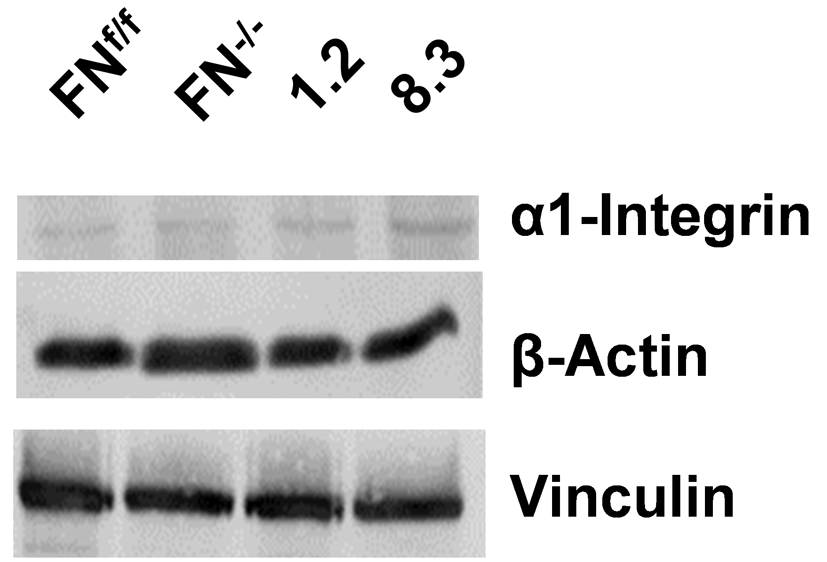

Supplement: FIGURE S5 — Expression level of integrin-α1 is very low but similar between all cell lines. Immunoblot of cell extracts obtained from FNf/f, FN−/−, clone 1.2, and clone 8.3 cells. Blots were probed with antibodies to integrin-α1, and with β-actin and vinculin for loading control. [file Image_5.TIF]

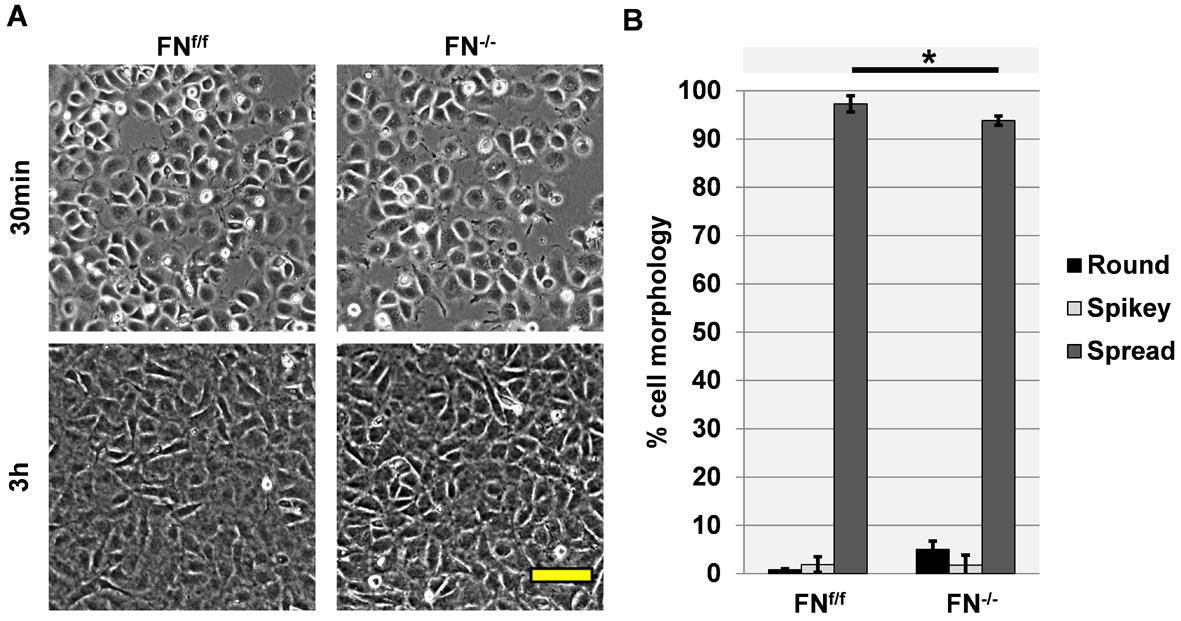

Supplement: FIGURE S6 — Spreading of FNf/f and FN−/− fibroblasts on FN-containing fibrin gels 3 h after seeding. (A) Representative images taken 30 min and 3 h after seeding cells (Scale bar: 100 μm). (B) The graph indicates the ratio in percentage (±SD) of “round” (black bars), “spikey” (light gray bars) and “spread” (dark gray bars) cells relative to the total number of cells. Statistical evaluation includes the average percentage of spread cells from three independent measurements (∗p < 0.05, unpaired t-test). [file Image_6.TIF]

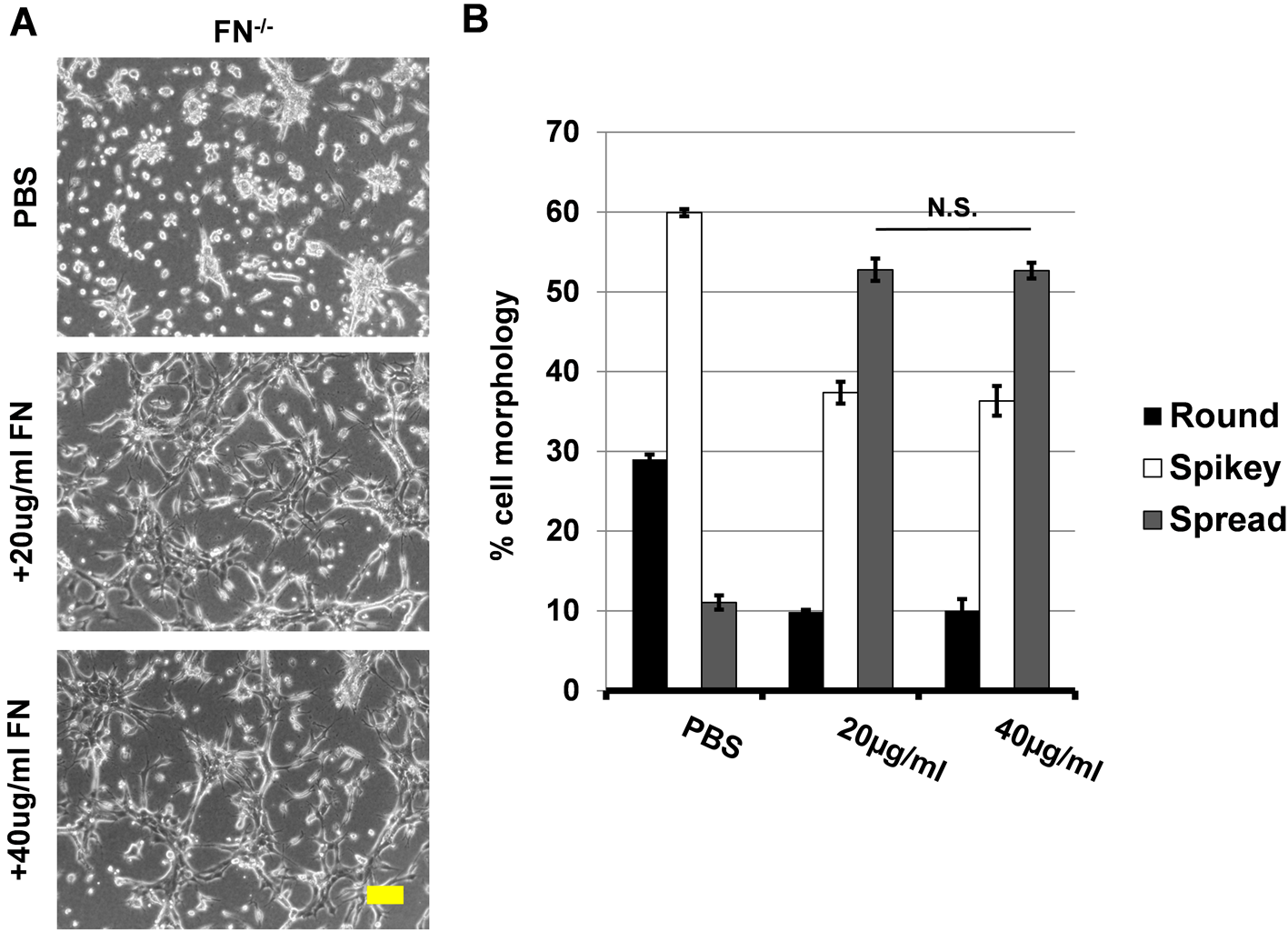

Supplement: FIGURE S7 — Doubling exogenous FN concentration does not further increase cell spreading of FN−/− fibroblasts on a 3D collagen matrix. (A) FN−/− cells require addition of 20 μg/ml exogenous FN to partially spread on collagen gel. Higher FN concentration (40 μg/ml) did not further enhance this behavior (Scale bar: 100 μm). (B) The ratio of spread FN−/− cells is statistically not significantly different (n.s.) between the two concentrations (20 or 40 μg/ml) of exogenous FN (±SD of three independent measurements, paired t-test). [file Image_7.TIF]

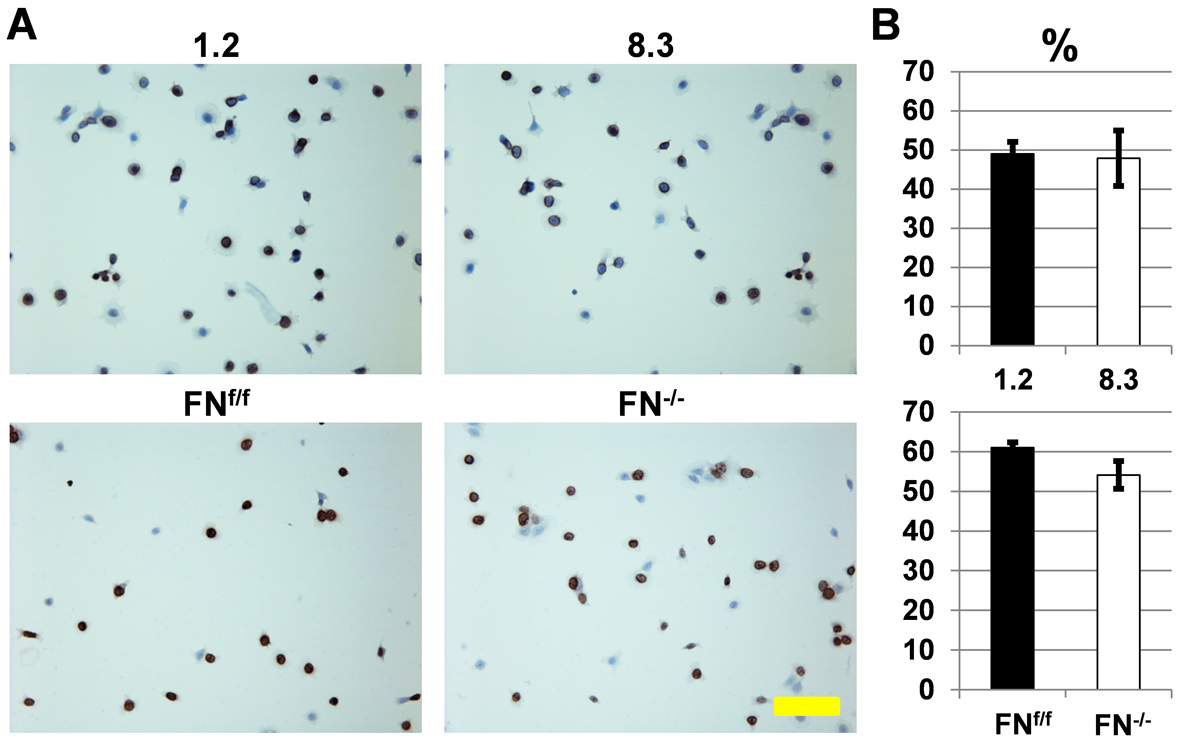

Supplement: FIGURE S8 — Similar proliferation rates of FN-deficient and control fibroblasts. (A) Representative images of BrdU-labeled cells, revealing newly synthesized DNA (brown) in a fraction of them. Hematoxylin (blue) was used as counterstaining. (B) Mean ratio of BrdU-positive cells relative to unstained (blue) cells as a percentage (±SD of three independent measurements, unpaired t-test) showed no difference between FN-deficient and control fibroblasts (Scale bar: 200 μm). [file Image_8.TIF]

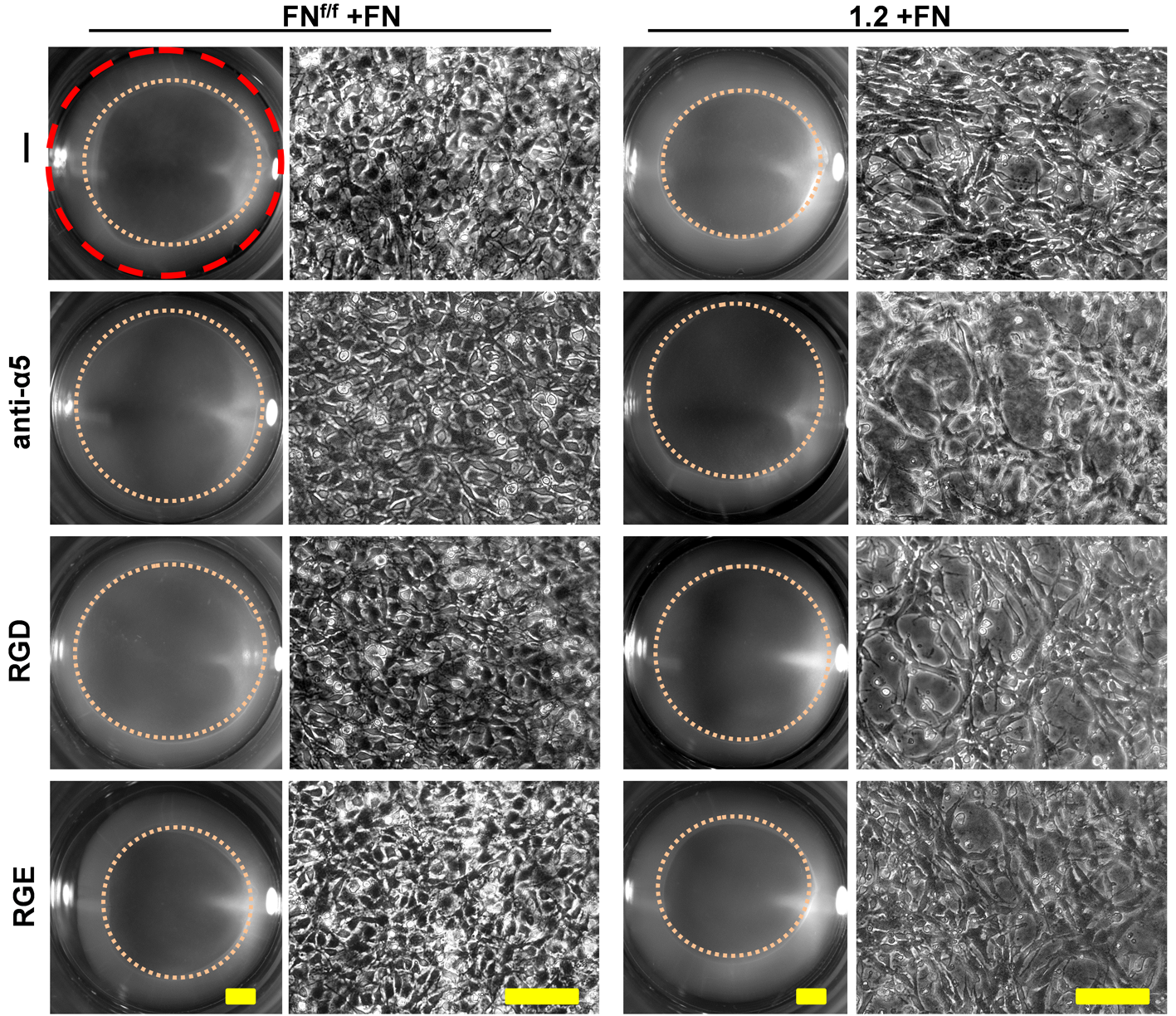

Supplement: FIGURE S9 — Impeded collagen gel contracture by FNf/f and 1.2 fibroblasts after adding function-blocking anti-integrin α5 antibody or RGD peptide. Representative images of contracted collagen gels (left panels; scale bar: 2 mm) and the respective spreading behavior FNf/f and 1.2 fibroblasts after 24 h (right panels; scale bar: 100 μm). Cultures were either left untreated (−), or alternatively a function blocking antibody against integrin α5 (anti-α5, 10 μg/ml), adhesion-blocking peptide GRGDSP (RGD, 1 mM), or control peptide GRGESP (RGE, 1 mM) were added to the medium. Collagen gel contraction was impeded after blocking integrin α5 by the respective antibody or by GRGDSP. Cell spreading was not affected by either inhibitor (see Figure 7 for quantification). [file Image_9.TIF]
